# Case report: Dermatosis neglecta mimicking pemphigus foliaceus in association with obsessive–compulsive disorder

**DOI:** 10.3389/fmed.2023.1076397

**Published:** 2023-03-23

**Authors:** Kantarat Wattanawinitchai, Poonkiat Suchonwanit

**Affiliations:** Division of Dermatology, Faculty of Medicine, Ramathibodi Hospital, Mahidol University, Bangkok, Thailand

**Keywords:** OCD, psychiatric management, psychodermatology, self-neglect, skin, self-cleaning, dermatitis neglecta, terra firma-forme dermatosis

## Abstract

Dermatosis neglecta (DN) is a frequently underrecognized skin disorder that occurs due to unconscious or conscious neglect of self-cleaning, causing the accumulation of keratin, sweat, sebum, and impurities. It is characterized by asymptomatic yellowish-to-brownish, waxy, adherent papules or plaques with cornflake-like scales that mimic several dermatological conditions. DN awareness is crucial for avoiding unnecessary invasive diagnostic procedures. Its lesions can be removed with some difficulty by ordinary cleansing and efficiently cleared with ethyl or isopropyl alcohol. Individuals with underlying physical or mental disabilities or psychiatric conditions are highly associated with DN. Nevertheless, supportive evidence for the coexistence of DN and psychological conditions is sparse, and most individuals with mental problems usually deny having psychiatric issues. Here, we present a case of DN resembling pemphigus foliaceus on the face with obsessive–compulsive disorder in a 16-years old male. The definitive diagnosis of DN in this patient was confirmed *via* histopathological examination. The lesions completely disappeared after appropriate facial cleansing and psychiatric management. DN may reveal underlying psychiatric disorders in patients.

## 1. Introduction

Dermatosis neglecta (DN) is an acquired asymptomatic skin disorder characterized by localized, yellowish-to-brownish, waxy, adherent, verrucous papules or plaques with cornflake-like scales that can be removed by washing with soap and water and completely cleared with ethyl or isopropyl alcohol (1). DN is considered a diagnostic challenge because of its clinical similarity to other skin disorders. It is associated with an act of omission, especially the neglect of self-cleaning, secondary to some underlying physical or psychological conditions (2). Due to the fact that the majority of patients frequently decline to have psychological problems, delaying psychiatric therapy, dermatologists are among the foremost clinicians to deal with them. Herein, we report the case of a patient with DN who presented with crusted lesions on the face mimicking pemphigus foliaceus. Psychiatric evaluation revealed an associated obsessive–compulsive disorder (OCD) in this patient.

## 2. Case report

A 16-year-old male patient visited the dermatology outpatient department with his father, presenting with progressive yellowish-to-brownish lesions on his face without abnormal sensation for 1 year. The lesions gradually enlarged in size and thickness and were located on both the cheeks and chin. He had no previous medical or mental problems, except for acne, which was self-treated with 2.5% benzoyl peroxide gel, 1% clindamycin lotion, sulfanilamide with tannin powder (an antibacterial herbal product), and a moisturizer; however, his acne was not considerably improved since he was 14. He also reported no family history of psychiatric illness. The patient could not remove the lesions with soap and wanted to cure this problem because of cosmetic concerns. He reported regular facial washing but was cleansing his face too softly because he felt disturbed by the use of force on his face. His father noticed his obsessively anxious feelings about face touching and concerns about the facial lesions. Accordingly, psychiatric consultation was performed during the first visit.

**Figure 1:**
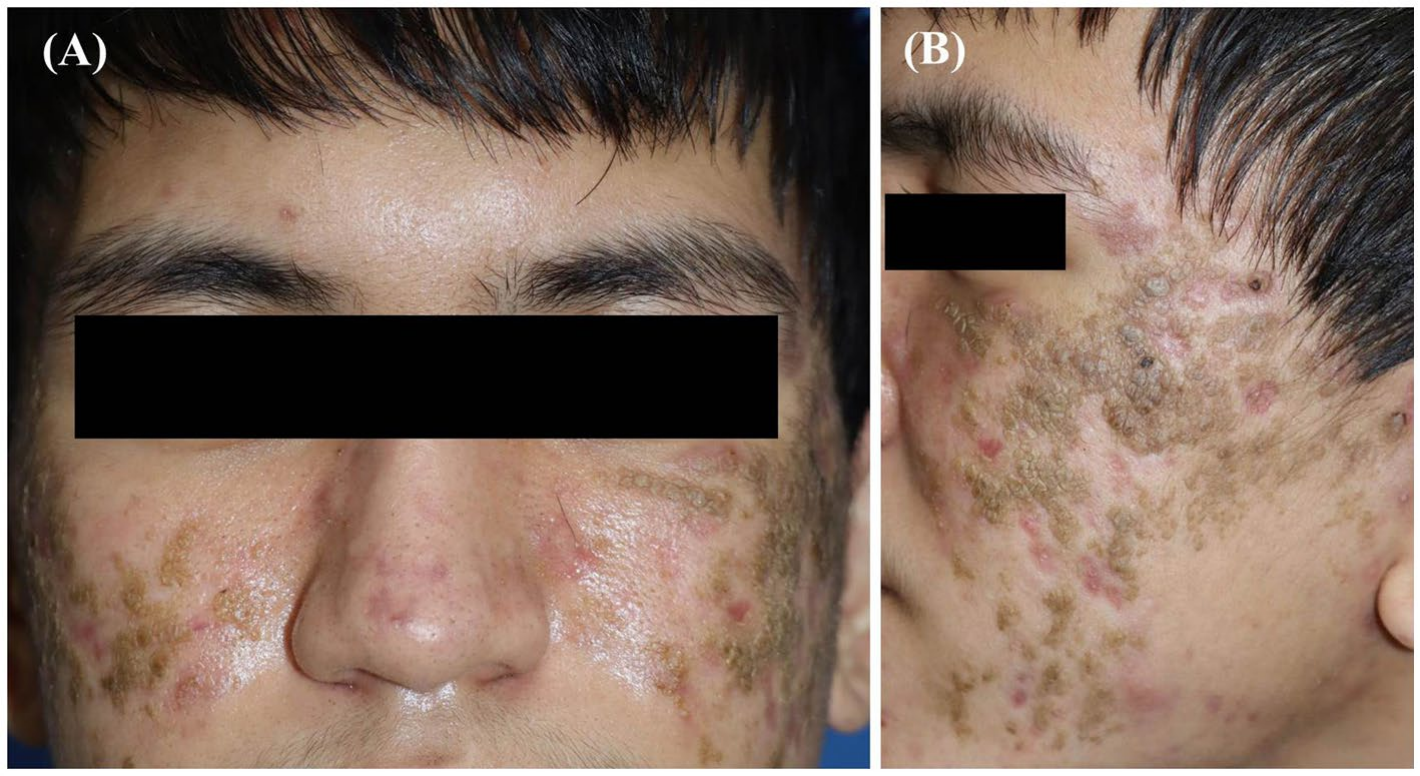
**(A, B)** Multiple well-defined yellowish-to-brownish crusted plaques with a verrucous surface on erythematous-based skin distributed on both cheeks.

**Figure 2:**
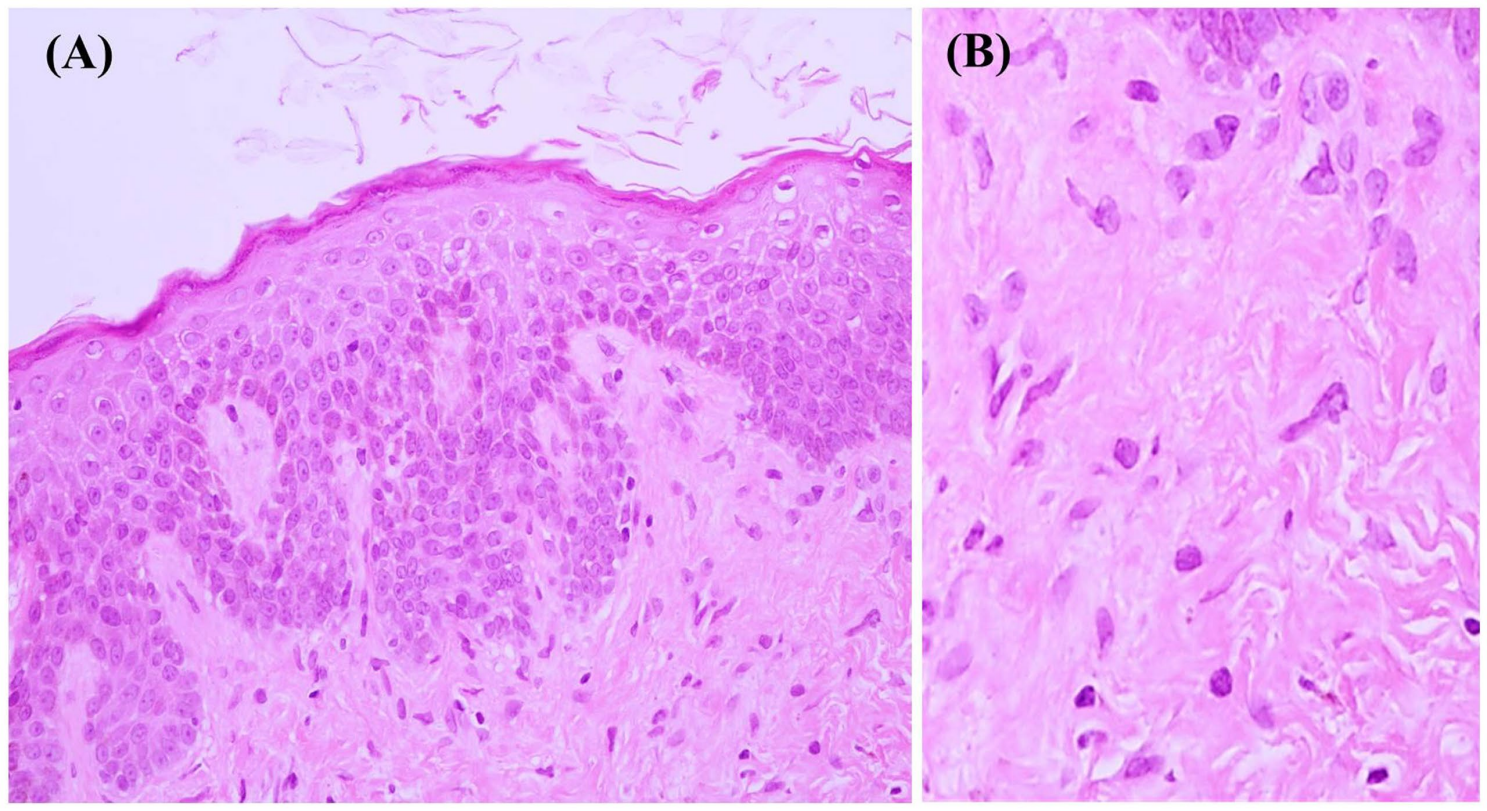
**(A)** Histopathological examination demonstrating orthokeratosis, mild epidermal spongiosis, and perivascular infiltration of lymphocytes in the upper dermis (hematoxylin–eosin; original magnification ×400); **(B)** High magnification of the dermal infiltrate (hematoxylin–eosin; original magnification ×1000).

Dermatological examination revealed multiple well-defined yellowish-to-brownish crusted plaques with a verrucous surface on erythematous-based skin distributed on both cheeks and chin (Figures 1A,B). Our differential diagnosis, in this case, included pemphigus foliaceus, DN, Darier’s disease, or seborrheic dermatitis. Based on suspicion of DN, wet compression with normal saline was performed on the lesions for 30 min. The majority of lesions were completely eliminated by rubbing with cotton swabs; however, a few remained. Then, we gently rubbed them with gauze and 70% ethyl alcohol, but the skin eroded and bled. After discussion with the patient, skin biopsy, routine blood tests, and anti-desmoglein 1 and 3 tests were performed for a definitive diagnosis. Histopathological findings revealed orthokeratosis, mild epidermal spongiosis, and perivascular infiltration of lymphocytes in the upper dermis (Figures 2A,B). Immunofluorescence study revealed negative results, and blood tests were within normal limits. Tests for anti-desmoglein 1 and 3 were also negative.

Regarding the psychological evaluation, the patient is an only child from a middle-class family and previously had no known psychological or mental health issues. There was no history of any learning disabilities. At the age of 12, the patient developed acne, which caused distress and diminished his confidence in his appearance. He began spending most of his free time playing computer games alone, neglecting his homework, and growing reluctant to attend school. He stayed up late at night and slept until the afternoon. However, his parents did not realize there was an issue and abandoned him because they also faced financial difficulties during the coronavirus disease 2019 pandemic. During the past year, the patient’s facial lesions became more noticeable. His father noticed he appeared frustrated and angry when other family members inquired about his facial lesions. As a result, he separated himself and disregarded his family. The diagnostic assessment using the Yale-Brown Obsessive Compulsive Scale revealed his score of 22, indicating moderate obsessive–compulsive symptoms. The patient’s compulsion to not touch his face appropriately was driven by his anxious thoughts, which caused him distress and clinically significant impairment in social interaction. Therefore, the diagnosis of OCD and social phobia secondary to his dermatological disorder was performed.

**Figure 3:**
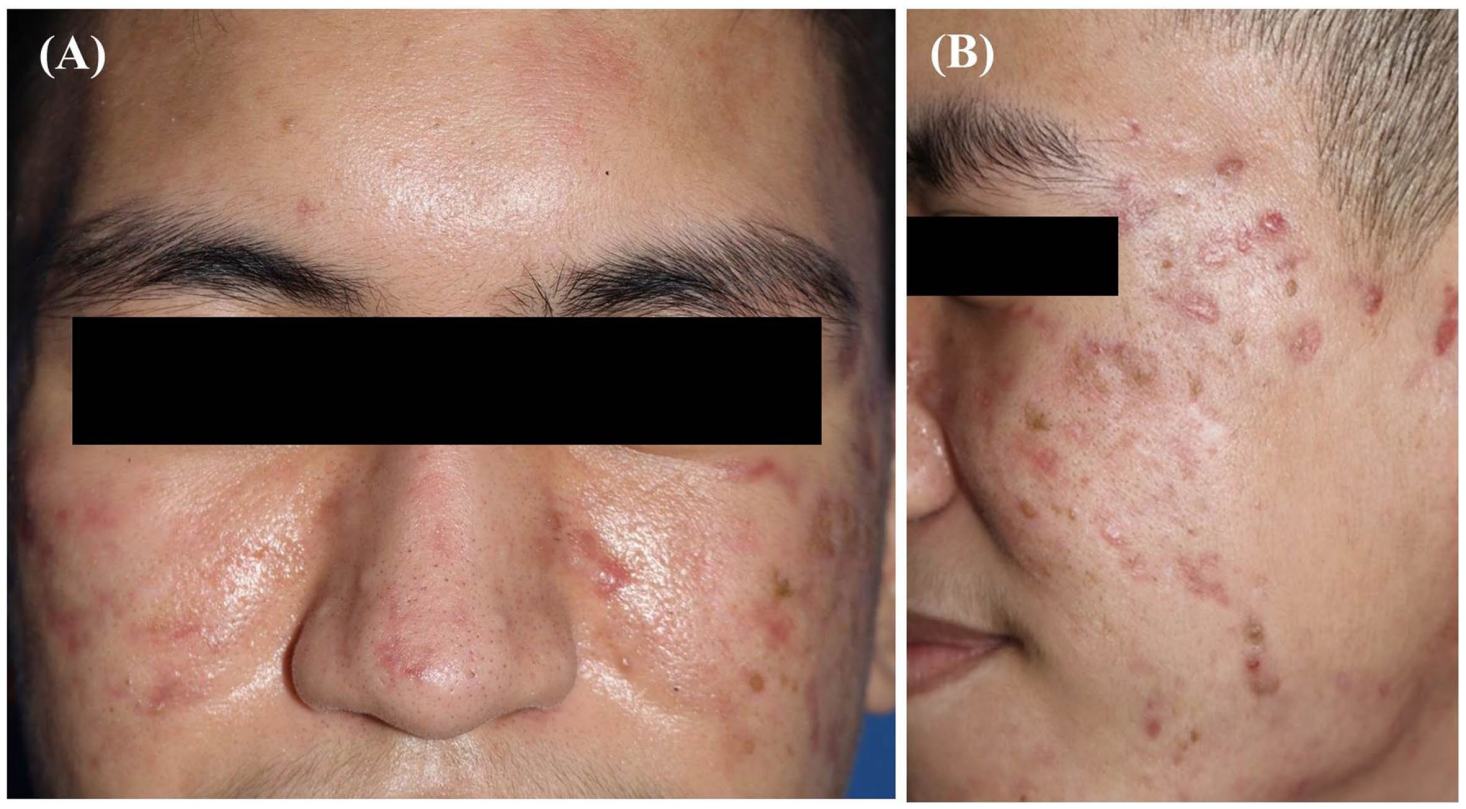
**(A, B)** The improvement of the lesions after 4 weeks of treatment.

Based on the clinical presentation, investigations, and psychiatric evaluation, the final diagnosis was DN. We advised and encouraged the patient on optimal facial care, using a mild cleanser, and gently rubbed the lesions with cotton swabs and either normal saline or 70% ethyl alcohol. The lesions were completely removed, leaving normal skin and atrophic acne scars. No recurrence was observed after 4 weeks of follow-up, confirming the DN diagnosis (Figures 3A,B). We prescribed oral doxycycline (200 mg/day), 2.5% benzoyl peroxide gel, 3% salicylic acid cream, and 1% clindamycin lotion for acne treatment and discussed further acne scar management. He was also treated with cognitive behavioral therapy (CBT) and fluoxetine (20 mg/day) for his psychiatric disorders. Exposure and Response Prevention techniques were used for his CBT. The psychologist instructed the patient to touch his face with appropriate force, assisted him in overcoming his fear of touching his face, and provided advice on managing his anxious thoughts and effectively implementing the techniques. At 3 months of treatment, considerable improvement in acne and mental problems was noted, and doxycycline and fluoxetine were discontinued.

## 3. Discussion

DN, also known as unwashed dermatosis, is a relatively newly introduced dermatological condition. Only a few case reports of DN have been published, but its prevalence is probably more common than that reported in the literature owing to misdiagnosis or underreporting (1). Poskitt et al. coined the term “dermatitis neglecta” in 1995 to describe a skin disorder secondary to lack of cleanliness in three patients (3). Later, Ruiz-Maldonado et al. changed the name to DN in 1999 due to the lack of inflammation in clinical presentation and histopathology (4).

DN generally occurs in all age and ethnic groups with no sex predilection (1). It is frequently underdiagnosed because it is asymptomatic, although pruritus and foul odors have been reported (3). Clinically, DN is characterized by brownish hyperkeratotic papules or plaques with cornflake-like scales, secondary to a lack of hygiene, and it generally takes 2–4 months to develop. Based on the reported cases, lesions can occur in all body areas and disappear with proper cleaning (5).

However, the pathogenesis of DN remains unclear. Progressive accumulation of keratinous debris, sebum, and sweat, forming a compact and adherent crust of dirt owing to inadequate skin cleaning, could explain the development of the lesions (6). Loss of self-care or hygiene maintenance, such as chronic disability, mental debilitation, motor or sensory impairment, immobility, post-surgery, hyperaesthesia, sensitive skin, or prior trauma, are considered risk factors for DN (1). The development of DN lesions in our patient could be initiated by the accumulation of sulfanilamide with tannin powder used for acne treatment and improper facial cleaning due to the patient’s discomfort with forceful face touching.

DN can be diagnosed by careful history-taking and physical examination. Patients with DN often accept a lack of cleanliness and act of omission of cleaning either intentionally or unconsciously (7, 8). The lesions can be removed with some difficulty by washing with soap and water and completely cleared with ethyl or isopropyl alcohol (1). DN can be frequently misdiagnosed because its clinical characteristics vary and mimic several dermatological disorders, e.g., seborrheic keratosis, verrucous nevi, psoriasis vulgaris, crusted scabies, and pigmented basal cell carcinoma (4, 6, 7, 9–13). Therefore, a skin biopsy is essential in some cases with overlapping manifestations. The histopathological features of DN are usually non-specific, commonly demonstrating a lack of inflammatory infiltrate, epidermal hyperkeratosis with orthokeratosis, acanthosis, and papillomatosis (6).

The differential diagnoses of DN include conditions with hyperkeratosis, papillomatosis, hyperpigmentation, and crusts, e.g., acanthosis nigricans, confluent reticulated papillomatosis of Gourgeot and Carteaud, Darier’s disease, epidermal nevus, and terra firma-forme dermatosis (TFFD) (14). The major disorder that must be distinguished from DN is TFFD, which has similar clinical manifestations and can be easily misdiagnosed for each other. TFFD or Duncan’s dirty dermatosis was first described by Duncan et al. in 1987 (15). Most patients with TFFD are older children and adolescents with normal washing habits, whereas DN affects patients of any age with inadequate scrubbing or cleaning of the skin, avoiding washing, or poor hygiene (16). TFFD is characterized by asymptomatic, brownish, hyperkeratotic, dirt-like papules or plaques resistant to washing with soap and water but is easily removed by rubbing with gauze immersed in alcohol (16).

The association between DN and psychiatric problems has been increasingly reported in the literature. These disorders usually manifest as obvious or part of the neglect of cleanliness. The reported conditions include OCD (as in our patient), mood disorders, affective disorders, catatonia, paranoia, schizophrenia, psychosis, intentional neglect for some secondary gain, intellectual disability, dementia, and substance use disorders (17–22).

The treatment of DN is based on reinforcing hygiene measures. Gentle rubbing with soap and water or saline-soaked moist gauze could be sufficient in all patients; however, forceful scrubbing with isopropyl alcohol or topical keratolytic agents, such as glycolic acid, salicylic acid, lactic acid, urea, or retinoids, is essential in severe cases (23–25). Proper psychological evaluation and management should also be considered in some patients.

## 4. Conclusion

DN is a skin disease that mimics other dermatological disorders and is frequently misdiagnosed. Loss of self-care or hygiene maintenance may be associated with this disease in some patients. Here, we report a biopsy-confirmed case of DN mimicking pemphigus foliaceus on the face with OCD that improved with appropriate facial cleansing and psychiatric management. Owing to its varied manifestations, physicians might be unaware of DN. Appropriate history-taking and simple removal of lesions with water, soap, or alcohol are essential to avoid unnecessary invasive diagnostic and therapeutic procedures. In addition, efforts should be made to identify and address underlying psychiatric conditions that benefit patients’ outcomes.

## Data availability statement

The original contributions presented in the study are included in the article/supplementary material, further inquiries can be directed to the corresponding author.

## Ethics statement

Written informed consent was obtained from the individual(s) for the publication of any potentially identifiable images or data included in this article.

## Author contributions

KW and PS were involved in direct management of the patient. KW drafted the first manuscript and reviewed the literature. PS reviewed and revised the manuscript. All authors contributed to the article and approved the submitted version.

## Conflict of interest

The authors declare that this manuscript was prepared in the absence of any commercial or financial relationships that could be construed as a potential conflict of interest.

## Publisher’s note

All claims expressed in this article are solely those of the authors and do not necessarily represent those of their affiliated organizations, or those of the publisher, the editors and the reviewers. Any product that may be evaluated in this article, or claim that may be made by its manufacturer, is not guaranteed or endorsed by the publisher.
